# Polarization-tuned Dynamic Color Filters Incorporating a Dielectric-loaded Aluminum Nanowire Array

**DOI:** 10.1038/srep12450

**Published:** 2015-07-27

**Authors:** Vivek Raj Shrestha, Sang-Shin Lee, Eun-Soo Kim, Duk-Yong Choi

**Affiliations:** 1 Department of Electronic Engineering, Kwangwoon University, 20 Kwangwoon-ro, Nowon-Gu, Seoul 139-701, South Korea; 2 Laser Physics Centre, Research School of Physics and Engineering, Australian National University, ACT 2601, Australia

## Abstract

Nanostructured spectral filters enabling dynamic color-tuning are saliently attractive for implementing ultra-compact color displays and imaging devices. Realization of polarization-induced dynamic color-tuning via one-dimensional periodic nanostructures is highly challenging due to the absence of plasmonic resonances for transverse-electric polarization. Here we demonstrate highly efficient dynamic subtractive color filters incorporating a dielectric-loaded aluminum nanowire array, providing a continuum of customized color according to the incident polarization. Dynamic color filtering was realized relying on selective suppression in transmission spectra via plasmonic resonance at a metal-dielectric interface and guided-mode resonance for a metal-clad dielectric waveguide, each occurring at their characteristic wavelengths for transverse-magnetic and electric polarizations, respectively. A broad palette of colors, including cyan, magenta, and yellow, has been attained with high transmission beyond 80%, by tailoring the period of the nanowire array and the incident polarization. Thanks to low cost, high durability, and mass producibility of the aluminum adopted for the proposed devices, they are anticipated to be diversely applied to color displays, holographic imaging, information encoding, and anti-counterfeiting.

Metal-dielectric nanostructures and nanoparticles that exhibit extraordinary light manipulating capability based on optical resonance have been extensively utilized to embody structural color filters, which are regarded as a central component in digital display and imaging devices, colorful decoration, anti-counterfeiting, and so on^1^,^2^,^3^,^4^,^5^,^6^. Various types of one-dimensional (1D)^7^,^8^,^9^ and two-dimensional (2D)^10^,^11^,^12^,^13^,^14^ nanostructures have been taken advantage of in order to realize a variety of spectral filters based on plasmonics in aluminum (Al) metal, taking into account its optimistic properties over commonly used plasmonic materials like gold (Au) and silver (Ag)^15^,^16^,^17^,^18^,^19^,^20^. In particular, the prominent aspects of Al encompass its natural abundance, neutral tint, good adhesion to diverse platforms, and excellent compatibility with the prevalent complementary metal-oxide-semiconductor (CMOS) process^15^,^16^,^17^,^18^,^19^,^20^,^21^,^22^. So far, structural color filters were mostly developed to produce one fixed color for a given geometry for incident white light. Dynamic color filters that enable adaptive color-tuning over a broad spectral range may be principally essential to diverse applications, such as ultra-fast displays, multi-spectral imaging, dynamic holographic imaging, information encoding, and stereoscopic color printing^23^,^24^,^25^,^26^,^27^,^28^. Thanks to the capability of 2D asymmetric nanostructures to excite surface plasmons (SPs) along two orthogonal directions depending on incident polarization, color filters relying on specially engineered 2D geometries were previously reported to generate polarization-dependent color output^26^,^27^. Implementation of such polarization-tuned dynamic coloration via simple subwavelength structures, like a 1D metallic nanowire (NW) array, without resorting to sophisticated geometries that require critical considerations in design and fabrication, remains a challenge, considering the fact that SP modes are known to be only supported for transverse magnetic (TM)-polarized light with the electric field oriented perpendicular to the metallic NWs while the optical transmission is significantly prohibited for transverse electric (TE)-polarized light. This is believed to be the primary reason why studies on the color filters that are based on a 1D metallic NW array are mainly focused on the generation of colors merely for the TM polarization, while little attention is paid to the TE case where the transmission is usually low and no distinct color is available^7^,^8^,^9^,^28^,^29^.

It should be remarked that production of two independent colors for both polarizations is an integral prerequisite for polarization-assisted dynamic color filtering. Colors may be generated through such 1D metallic NW arrays in a subtractive scheme by tapping into the SP resonance (SPR) under the TM incidence, exploiting the wavelength-selective suppression of transmission^30^. For the TE case, however, creation of subtractive colors via such 1D nanostructures poses two paramount issues: firstly, achieving high transmission across a broad spectral range, and, secondly, inhibition of transmission in the proximity of specific wavelengths. The former requirement might be satisfied by filling up the slits of the NW array with a dielectric in order to increase the cutoff wavelength and support cavity modes in the slits as previously reported^31^,^32^, while the latter can be potentially addressed by virtue of other non-plasmonic phenomena, like the guided-wave resonance (GMR)^33^. Such 1D NW array for mitigating those two issues can enable enhanced transmission for both TE and TM polarizations, providing independent control of the locations in relation to suppressed transmissions for the two polarizations.

To date, no attempt has been made towards the embodiment of polarization-controlled dynamic color filters that incorporate a 1D metallic NW array. In this work, we propose and accomplish highly efficient polarization-tuned dynamic color filters resting on a dielectric-loaded 1D aluminum (Al) NW array, by appropriately capitalizing on the SPR in conjunction with the GMR. We are particularly concerned about the widely tunable color filters drawing upon a 1D Al NW array, the slits of which are fully filled with a dielectric of SiO_2_ along with an appropriately thick overlay of the same dielectric. The proposed filters are capable of producing a continuum of colors that is represented by a line on the chromaticity diagram, delivering the customized color output in accordance with the polarization of incident light, unlike conventional color filters, which just produce a single fixed color that is represented by a point on the CIE 1931 chromaticity diagram. The filters exploit the SPR and GMR under the TM and TE polarizations, respectively, in order to manifest selectively suppressed transmissions at different spectral positions, thereby enabling the generation of double distinct colors for the two orthogonal polarizations. For the state of polarization intermediate between the two extremes of TM and TE, the color lying between the two distinct colors would be generated, signifying that the optical output can be continuously tailored across a wide range of color, by simply adjusting the polarization of input white light. The mechanism responsible for such polarization-dependent dynamic color filtering has been meticulously scrutinized by exploring the origin of the polarization-selective suppressed transmission that occurs at different wavelengths. The proposed nanostructure-based dynamic color filters can be readily applied to ultra-high-resolution displays, security tags, anti-counterfeiting, and steganography^26^,^27^.

## Results

### Subtractive Color Filters Featuring Polarization-tuned Transmission Spectra

**Figure 1:**
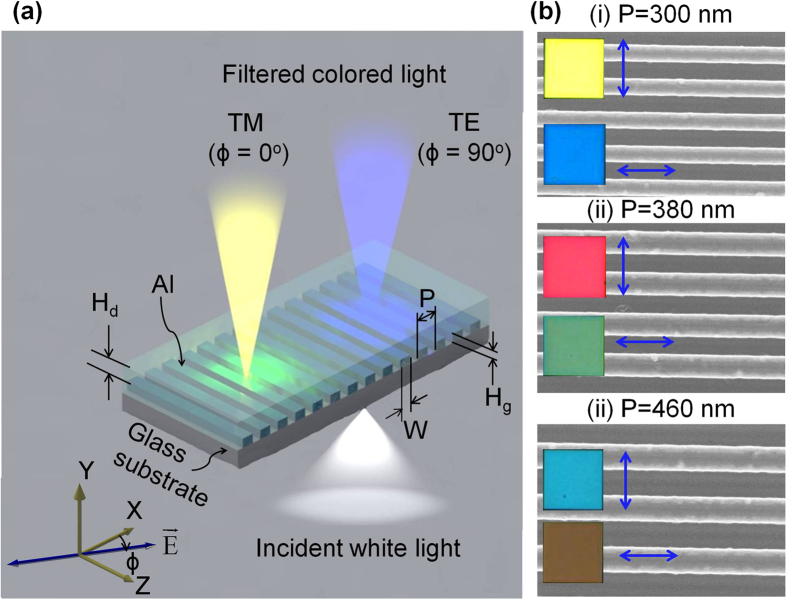
Dynamic color filters comprising of a dielectric-loaded Al 1D NW array. (**a**) Schematic diagram of the proposed dynamic color filters comprising of dielectric-loaded Al 1D NW array over a glass substrate. Incident white light is filtered into different visible colors, in accordance with the polarization (ϕ) of the incident light for the filter with a given period P. (**b**) SEM images of the fabricated filters with different periods of (i) 300, (ii) 380, and (iii) 460 nm, from top to bottom, including their generated color images in the inset for different polarization directions as indicated by the direction of E-field (blue arrow). It can be seen that the colors switched between yellow-blue, magenta-green and cyan-brownish red respectively for the two orthogonal polarizations, for the filters with P = 300, 380, and 460 nm, respectively.

The proposed dynamic color filter, whose schematic is illustrated in Fig. 1(a), consists of a 1D Al NW array, the slits of which are completely packed with dielectric (SiO_2_) and is covered with a thin overlay of the same dielectric. The metallic NW array has a thickness of H_g_ (=120 nm), a period P along the *x*-direction, and a duty ratio of 0.5, which is defined as the ratio of the width (W) of each NW to the period (P). The dielectric overlay has a thickness of H_d_ (=200 nm). The alignment of the electric field (E-field) with respect to the *x*-direction in the *xz*- plane, which is indicated by an angle ϕ, determines the polarization state of the incident light. The light transmission through the filter may be selectively hindered at specific resonance wavelengths in accordance with ϕ, so as to produce adaptively varying colors. For instance, as depicted in Fig. 1(a), incoming white light is efficiently filtered out to give rise to yellow color for ϕ = 0°, i.e. for the TM polarization, while blue color is obtained for ϕ = 90°, i.e. for the TE polarization. The dielectric overlay not only serves as a protective layer against environmental factors but also acts as a functional layer for dynamic color generation, which will be discussed later in this paper and the Supplementary Information. Also, it will be addressed later that, for the TM polarization, the filtering of visible light to produce specific colors is acquired through the SPR, resulting in selective suppression of transmission while, for the TE polarization, the generation of color is ascribed to the resonance of guided modes supported by a leaky planar waveguide, which is formed by a core of the dielectric overlay that is surrounded by claddings of the metallic NW array and air.

Figure 1(b) displays scanning electron microscope (SEM) images of the prepared dynamic color filters with periods of 300, 380, and 460 nm, respectively, from top to bottom. The generated bright color images with high contrast are included in the inset, for different polarizations as indicated by the E-field (a blue arrow). The created Al NW pattern associated with the filters was observed under a high-resolution SEM (UltraPlus analytical FESEM from Zeiss) while the corresponding color images were captured with a digital color microscope camera (Leica DFC450), installed to a digital microscope (Leica DM4000 M). For the filters with P = 300, 380, and 460 nm, the color output appeared to switch between the pairs of yellow and blue, magenta and green, and cyan and brownish red; the former and latter colors of those pairs being respectively generated for the two orthogonal polarizations, TM and TE. For the dynamic color filters that were formed on a glass substrate with dimensions of 40 μm × 40 μm, a well-defined 1D NW array has been well manufactured, exhibiting high fidelity according to the design, as revealed in the SEM images.

**Figure 2:**
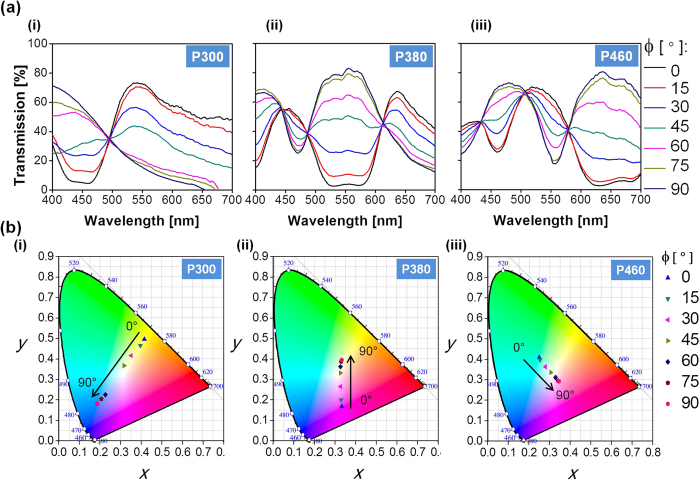
Polarization-tuned transmission spectra and corresponding color responses. (**a**) Measured transmission spectra of the filters with NW array periods of (i) 300, (ii) 380 and (iii) 460 nm, respectively, from left to right respectively, for different polarization directions from ϕ = 0° to 90° in the steps of 15°. Transmission spectra were highly affected by incident polarization, indicating the polarization-tunability. (**b**) Chromaticity coordinates corresponding to the measured spectra for filters with periods of (i) 300, (ii) 380, and (iii) 460 nm in the CIE 1931 chromaticity diagram as polarization angles ϕ varied from 0° to 90° in steps of 15°.

For the prepared filters with NW periods of 300, 380, and 460 nm, the measured transmission spectra are respectively depicted in Fig. 2(a) (i), from left to right, under different polarizations varying from ϕ = 0° to 90° in steps of 15°. High transmission surpassing 80% in the visible regime was attained for both TM (ϕ = 0°) and TE (ϕ = 90°) polarizations. For the TM case, the filters with periods of 300, 380, and 460 nm had a broad transmission dip centered at λ = ~450, 550, and 650 nm, respectively. Meanwhile, for the TE polarization, a low-pass transmission characteristic was observed for the filter with P = 300 nm. It was found for the other two devices with P = 380 and 460 nm that the low-pass spectral response was locally affected by relatively sharp dips centered at λ = 466 and 540 nm, respectively. It was confirmed theoretically and experimentally that, for other intermediate polarization directions residing between ϕ = 0° and 90°, the transmission was determined by the combination of the transmissions in relation to the two orthogonal polarizations. That is, when the transmissions for orthogonal cases of ϕ = 0° and 90° were designated as T_0_ and T_90_, the transmission for an arbitrary polarization of ϕ was given by T_*ϕ*_ = T_0_cos^2^*ϕ* + T_90_sin^2^*ϕ*^26^. Supplementary Figure S1(a) shows the corresponding calculated spectra for the filters with NW periods of 300, 380, and 460 nm, which were obtained by use of a simulation tool based on the finite difference time domain (FDTD) method (FDTD Solutions, Lumerical, Canada). The good agreement between the simulation and the measurement results was confirmed from Fig. 2 and Supplementary Figure S1.

### Dynamic Color-tuning and Demonstration of a Broad Palette of Subtractive Colors

For the proposed color filters, in a bid to have an insight into the color response in terms of the polarization, the chromaticity coordinates in response to the measured and simulated transmission spectra, as shown in Fig. 2(a), were calculated by using standard equations^34,35^ and plotted in a standard CIE (International Commission on Illumination) 1931 chromaticity diagram, as illustrated in Fig. 2(b). The chromaticity coordinates for the measured spectra of the filters with P = 300, 380, and 460 nm under different polarization angles, varying from ϕ = 0° to 90° in steps of 15°, are depicted in Fig. 2(b) (i) through (iii), respectively. The coordinates for the corresponding simulated spectra are plotted in Supplementary Figures S1 (b) (i) through (iii). In view of the variations in the chromaticity coordinates as a function of the angle ϕ, the color emerging from each of the filters can be dynamically customized by altering ϕ, as marked by the black arrow. For the device with P = 300 nm, the acquired color was tuned from yellow towards blue when ϕ changed from 0° to 90°, as marked by the coordinates in the diagram. Similarly, as ϕ varied from 0° to 90°, the output color that was available from the filter with P = 380 can be tuned across magenta and green. For the case with P = 460 nm, the output color was scanned between brownish red color and cyan. The coordinates for the simulated and measured spectra offered good correlations. Practically, factors, including surface roughness, fabrication defects, and non-parallel incident light used for measurements, which were not fully considered in the simulations, might account for the difference in absolute levels of the spectra, thus resulting in slight discrepancies between the chromaticity coordinates for the calculated and measured spectra.

**Figure 3:**
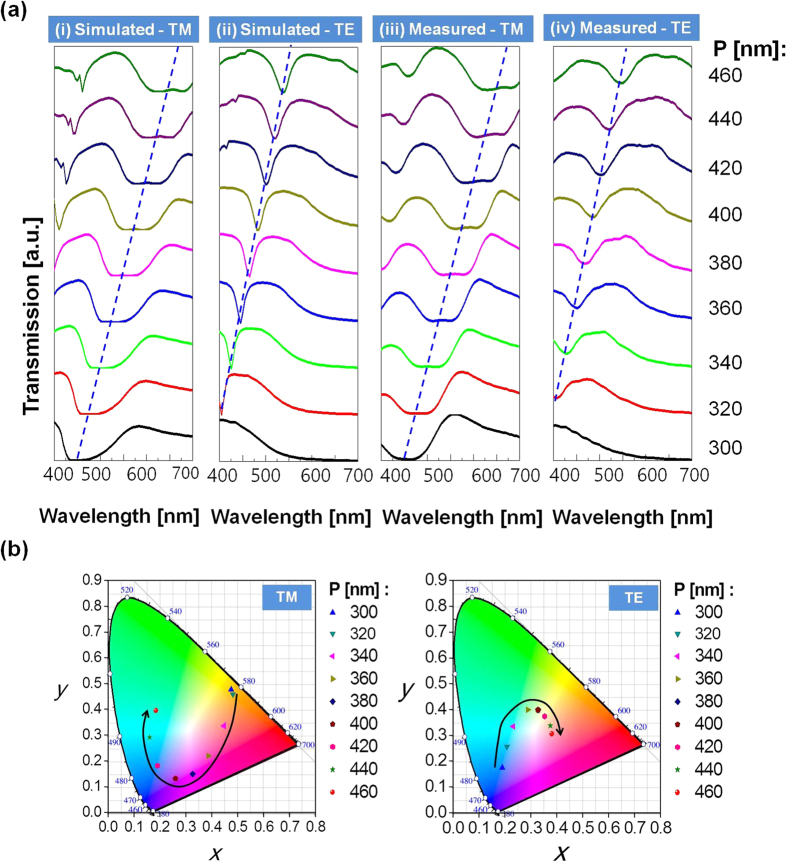
Period-dependent transmission spectra and corresponding color responses for the TM and TE incident polarizations. (**a**) Simulated and measured transmission spectra of the filters for TM (ϕ = 0°) and TE (ϕ = 90°) incident polarizations when the NW array period was varied from 300 to 460 nm. As observed in both simulation and measurements, transmission dips were witnessed in the case of both TM and TE incident polarizations, as indicated by dashed trend line tracing the location of the suppressed transmission. (**b**) Variation of the CIE 1931 chromaticity coordinates corresponding to the measured transmission spectra of the filters with different periods of the NWs for TM and TE polarizations, when the NW array period was varied from 300 to 460 nm in steps of 20 nm.

In an effort to assess the effect of the period of the NW array upon the performance of the dynamic color filters, the transmission spectra with respect to TM and TE polarizations were scrutinized through FDTD based simulations. Figure 3(a) shows the simulated and measured spectra for an Al NW array for the TM (ϕ = 0°) and TE (ϕ = 90°) polarizations, with the pitch ranging from 300 to 460 nm in increments of 20 nm, with the duty ratio fixed at 0.5. For the TM case, the optical response presented a broad dip according to the period, following a trend designated by the dashed line, where the position of the diminished efficiency shifted from λ = 450 to 650 nm in an approximately linear manner, with the pitch varying from 300 to 460 nm. Meanwhile, for the TE case, the response exhibited a dip dependent upon the period, following a trend indicated by the dashed lines, where the position of the prohibited transmission almost linearly shifted from λ = 420 to 538 nm, with the pitch varying from 320 to 460 nm. For a pitch of 300 nm, the dip lay in the UV regime below the visible band, which is hidden in Fig. 3(a). The measured characteristics for both TM and TE polarizations revealed a profoundly lowered transmission along a dashed trend line, closely resembling that of the simulated results. It may be inferred from the linear shift in the spectral location of dips with respect to the polarization that a broad palette of colors can be concocted by altering the period P.

We then corroborated that the period of the NW array can be tailored to demonstrate independent palette of colors under TM and TE polarizations. Toward that end, for filters with various periods, the chromaticity coordinates according to the measured responses under TM and TE incidences were plotted in a standard CIE 1931 chromaticity diagram, as illustrated in Fig. 3(b), while the coordinates for the simulated responses are illustrated in Supplementary Figure S2. The chromaticity coordinates evolved along the contour with increasing P, as indicated by the black curved arrow, implying that, besides the polarization angle ϕ, the structural parameter P can become another degree of freedom in customizing the color output. As observed from Fig. 3(b), the output colors for the filter with a constant P were not the same, suggesting that the output color for a filter with a given period P may be adaptively tuned contingent upon the polarization. An appreciable degree of agreement has been witnessed between the chromaticity coordinates for simulated and measured spectra, except for the slight discrepancies as aforementioned.

**Figure 4:**
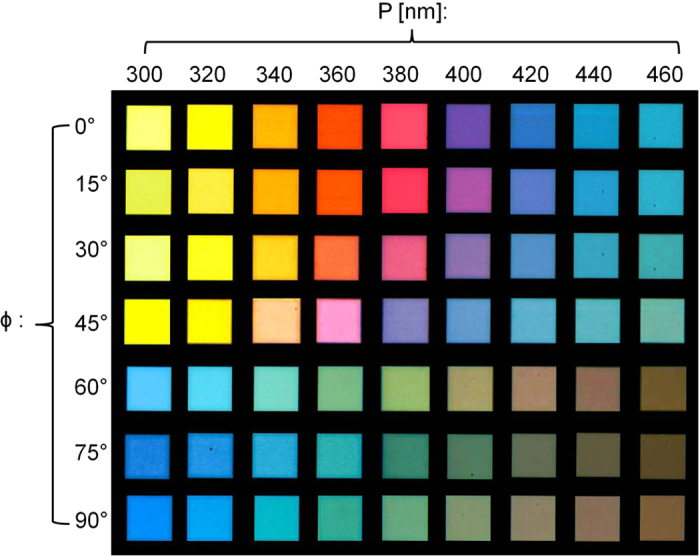
Optical micro-photographs in accordance with the period of NW array and incident polarization. Transmission-mode optical micro-photographs of the 40 μm × 40 μm sized filters, where the corresponding images, as the period of the NW array was varied from 300 to 460 nm in steps of 20 nm, are arranged along the column, while the images for each period of the filters, as the polarization angle ϕ was altered from 0 to 90° in increments of 15°, are arranged along the row.

In order to verify that different colors can be generated in accordance with the period of the NW array and the incident polarization, we have captured optical micro-photographs of the completed dynamic color filters. Figure 4 presents the transmission-mode images of the color output from the devices, where the corresponding images, as the period of the NW array was varied from 300 to 460 nm in steps of 20 nm, are arranged along the column, while those of the filters for each period, as the polarization angle ϕ was altered from 0° to 90° in steps of 15°, are arranged along the row. A wide variety of bright colors that featured high contrast appeared to be realized in terms of the polarization as well as the periodicity of the NW array. Consequently, the proposed dynamic color filters are apparently anticipated to outstrip their conventional cases in regard to the operational range, thereby expediting their applications to the display and multispectral imaging^30^.

It is to be mentioned that with an objective of creating a polarization-tuned dynamic color filter, which gives rise to polarization-selective distinct suppressed transmission at a certain spectral band, we have probed the effect of the major structural parameters, such as the thicknesses and the duty ratio of the NW array, in addition to the thickness of the dielectric overlay. It was discovered that, for a 120-nm thick NW, a duty ratio of 0.5, and a dielectric overlay of 200 nm, subtractive colors could be successfully produced by ensuring a negligibly small transmission at resonance wavelengths, yet guaranteeing high transmission efficiency over spectral bands apart from the resonance. Filling of the slits between the metallic NWs helped enhance the transmission for both TM and TE polarizations while the adoption of a dielectric overlay with an optimum thickness allowed transmission dips to be formed at the corresponding characteristic wavelengths, thereby making it possible to generate colors under both polarizations. Details on the transmission and color responses as obtained from the different thicknesses of dielectric coatings have been addressed in the Supplementary Figures S3 through S6.

### Mechanism for Polarization-tuned Selective Transmission-dips

**Figure 5:**
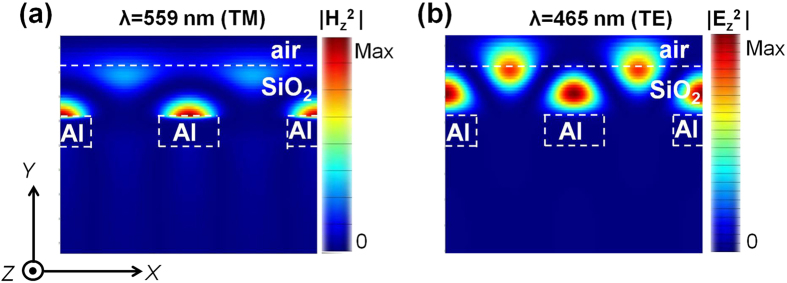
Field intensity plots at the location of transmission dips. 2D color maps of the z-component of (**a**) the magnetic field intensity (|H_z_^2^|) at λ = 559 nm for TM incidence and (**b**) the electric field intensity (|E_z_^2^|) at the location of the dip at λ = 465 nm for the TE incidence, through a vertical cross-section of two unit cells of the dielectric-loaded Al NW array over a glass substrate.

In an attempt to better understand the core physical mechanism underlying the selective spectral dips in transmission for TM and TE polarizations, we have inspected the near field profiles at the locations of transmission dips. As shown in the simulated graphs of Fig. 3(a), for a typical case of NW array with P = 380 nm, the locations of near-zero transmission dips were at wavelengths of 559 and 465 nm, respectively, for the TM and TE polarizations. For the TM case, we examined the z-component of the magnetic-field intensity distribution through a vertical cross-section of two unit cells of the dielectric-loaded Al NWs with a 380-nm period. As displayed in Fig. 5(a), for the transmission dip located at λ = 559 nm, the magnetic field was mostly confined at the metal-dielectric top interface, signifying the presence of SP modes, which were accountable for the substantially inhibited transmission leading to a wide plasmonic bandgap^36^,^37^. Similar field distributions were observed throughout the broad dip of negligible transmission. The z-component of the E-field intensity distribution was subsequently checked for the TE case. As displayed in Fig. 5(b), for the dip at λ = 465 nm the field was mainly confined in the dielectric overlay atop the Al NW. The field of concern was scarcely seen to penetrate into the Al NW towards the substrate, causing a relatively narrow dip in transmission. Actually the field enclosed in the dielectric overlay was thought to represent a standing wave along the x-direction, hinting at the excitation of guided modes supported by a dielectric planar waveguide^38^,^39^. By virtue of the resonance mediated by the guided modes, incoming light was reflected back towards the incident medium so as to bring up the dip in transmission^38^,^39^. Similar interpretations may be surely applied for other cases with different P.

**Figure 6:**
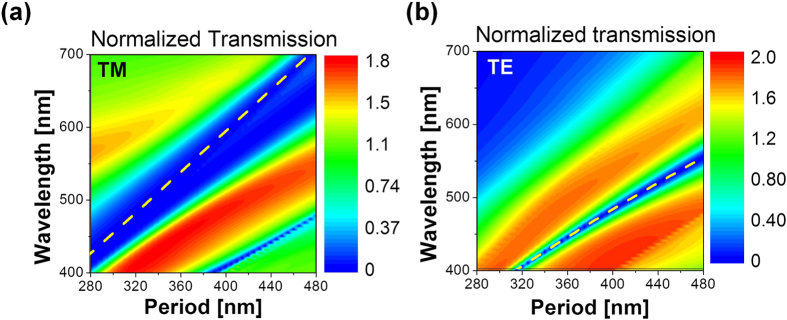
Transmission spectra with the dispersion of SP and guided modes included. Contour maps of the calculated transmission spectra that were normalized to the area of the slits for (**a**) TM and (**b**) TE incidence. The curves for the analytically derived dispersion relations both for the SPs at the Al-SiO_2_ interface and the guided modes supported by the air-SiO_2_-Al waveguide were superimposed on the contour maps, accurately predicting the location of the corresponding dips.

In order to validate the two principal phenomena of SPR and GMR that were presumed to play a central role in selectively prohibiting transmissions for the TM and TE incidence, respectively, we superimposed the curves corresponding to the analytically derived dispersion relation on the contour maps for the calculated spectra that were normalized to the area of the slits, as illustrated in Fig. 6(a,b). The analytical dispersion relation for the SPs excited at a metal-dielectric interface for the TM case is given by^40^:
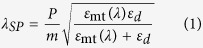
where *ε*_*d*_ and *ε*_mt_(*λ*) are the dielectric constants of the dielectric and metallic layers, respectively, and *m* is an integer signifying the order of resonance. As plotted in Fig. 6(a), the position of simulated transmission dips for the TM case shifted progressively from λ = 426 to 708 nm along the yellow dashed line as P increased from 300 to 480 nm, thereby supporting that the SP resonance was responsible for selectively inhibiting the spectral transmission. Meanwhile, as shown in Fig. 6(b), the location of the dips in the simulated spectra for the TE case, which evolved progressively from λ = 384 to 553 nm as P increased from 300 to 480 nm, can be accurately predicted by the yellow dashed line, representing the dispersion curve relating to the metal-clad planar waveguide of air-SiO_2_-Al configuration. It has been ultimately proven that the selectively low transmission for the TE polarization is categorically imputed to the presence of the GMR as predicted. For reference, the dispersion relation for the TE waveguide modes supported by such waveguide is known to be given by^41^:
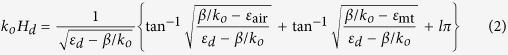
where *ε*_air_ is the dielectric constant of air, *k*_*o*_ = 2π/*λ*_*o*_, *λ*_*o*_ the free-space wavelength, *β* the propagation constant for the guided modes, and *l* an integer signifying the order thereof. The GMR is supposed to ensue when the phase matching condition is satisfied, requiring *β* to equal the grating vector G (=2 π/P)^42^.

## Discussion

In summary, we have taken advantage of a dielectric-coated 1D Al NWs built on a glass substrate so that we can present a new functional device capable of demonstrating highly efficient polarization-tuned dynamic color filtering with transmission beyond 80% for both TM and TE polarizations. It is based on selective suppression of transmission at characteristic wavelengths under the two polarizations, stemming from the SPR at the metal-dielectric interface and the GMR in the metal-air-dielectric waveguide under the TM and TE incidence, respectively. This was validated by keenly monitoring the near field profiles at the corresponding locations of different dips for the TM and TE cases. The theoretical prediction of the phenomena has also been addressed so as to support our discussions using the corresponding dispersion relations. We have presented detailed discussions on the role of the functional dielectric overlay atop the metallic NW array so that we could achieve dynamic color-tuning by means of simple structures, like a 1D metallic NW array, without resorting to special geometries of nanostructures. The filter provides us with two degrees of freedom so as to customize the transmitted colors in transmission mode, i.e. the periodicity of the NW array, which was pre-determined prior to fabrication, and the polarization of incident light, which rendered post-fabrication color-tuning possible. Based on the two degrees of freedom, a broad palette of colors has been demonstrated, including the primary subtractive colors of cyan, magenta and yellow. Despite the simple structure of devices with a NW array covered with an appropriately thick, a prominent functionality of dynamic color-tuning has been successfully demonstrated in this work, thereby eliminating the necessity of special geometries that demand special considerations to design and fabrication. Although polarization-tuned filtering has been mainly studied in this work, it should be stated that the structure was conceived as an interesting possibility in which the proposed device could be designed to make the TM and TE resonances occur at the same wavelength, thereby suggesting polarization insensitive spectral filtering characteristics^33^. Finally, coupled with the aforementioned distinctive features of Al in plasmonic or photonic applications, the proposed devices are anticipated to open an avenue towards further research and development of multi-functional dynamic color filters in Al.

## Methods

### Numerical Simulations

Simulations of the transmission spectra were performed by using a simulation tool based on the finite difference time domain (FDTD) method (FDTD Solutions, Lumerical, Canada) taking into account the complex refractive indices of Al and SiO_2_ from Palik^43^. Simulations were performed in the case of a plane wave under normal incidence. We employed a unit cell with periodic boundary conditions in order to practically mimic an infinite array of NWs. The calculation domain was divided with a non-uniform mesh refined with a conformal mesh technique, so that the calculation mesh size was as small as 4 nm^44^,^45^.

### Device fabrication

The proposed color filters were fabricated with dimensions of 40 μm × 40 μm. A 120-nm thick Al film was deposited on a glass substrate with an electron-beam evaporator (Temescal BJD-2000 E-beam/Thermal Evaporator system), which was subsequently patterned by means of an electron-beam lithography (EBL) system (RAITH 150) using a positive electron-beam resist of ZEP520A, and then dry etched in a plasma etcher (Versaline LL ICP Etching system) using a mix of Cl2, BCl3, and Ar gases. Then, a 200-nm thick SiO2 overlay was coated using plasma-enhanced chemical vapor deposition (Plasmalab 100, Oxford).

### Optical characterization

A high-resolution scanning electron microscope (SEM), (UltraPlus analytical FESEM from Zeiss) was used to observe the created Al pattern. In order to assess the transmission spectra under different incident polarizations, a calcite crystal polarizer (GTH 10M-A, Thorlabs, Operating range: *λ* = 350–700 nm) was used to appropriately polarize the collimated beam from a Xenon lamp (Model Super Bright 152S) and irradiated upon the prepared filters that were mounted on a motorized rotation stage, via a focusing objective lens, while the transmitted light was coupled to a spectrometer (Avaspec-3648, Avantes) via a multimode fiber. The transmission spectra of the filters were normalized with respect to the transmission of a bare glass. The color images of the filters were captured by a digital color microscope camera (Leica DFC450) attached to a digital microscope (Leica DM4000 M).

## Additional Information

**How to cite this article**: Raj Shrestha, V. *et al.* Polarization-tuned Dynamic Color Filters Incorporating a Dielectric-loaded Aluminum Nanowire Array. *Sci. Rep.* **5**, 12450; doi: 10.1038/srep12450 (2015).

## Supplementary Material

Supplementary Information
