# Supplementary material for: Polarization-tuned Dynamic Color Filters Incorporating a Dielectric-loaded Aluminum Nanowire Array
Source: Sci Rep. 2015 Jul 27;5:12450. doi: 10.1038/srep12450 (PMC4515642; doi:10.1038/srep12450)
Supplement: Supplementary Information [file srep12450-s1.doc]

SUPPLEMENTARY INFORMATION

Polarization-tuned Dynamic Color Filters Incorporating a Dielectric-loaded Aluminum Nanowire Array

Vivek R. Shrestha,^†^ Sang-Shin Lee*,^†^ Eun-Soo Kim,^†^ and Duk-Yong Choi^‡^

^†^Department of Electronic Engineering, Kwangwoon University, 20 Kwangwoon-ro, Nowon-Gu, Seoul 139-701, South Korea

^‡^Laser Physics Centre, Research School of Physics and Engineering, Australian National University, Canberra, ACT 2601, Australia

* E-mail: slee@kw.ac.kr

Subject terms: Physical sciences, Applied physics, Nanotechnology, Optical physics, Nanophotonics, Plasmonics

**1. Dependence of device performance on the incident polarization and period
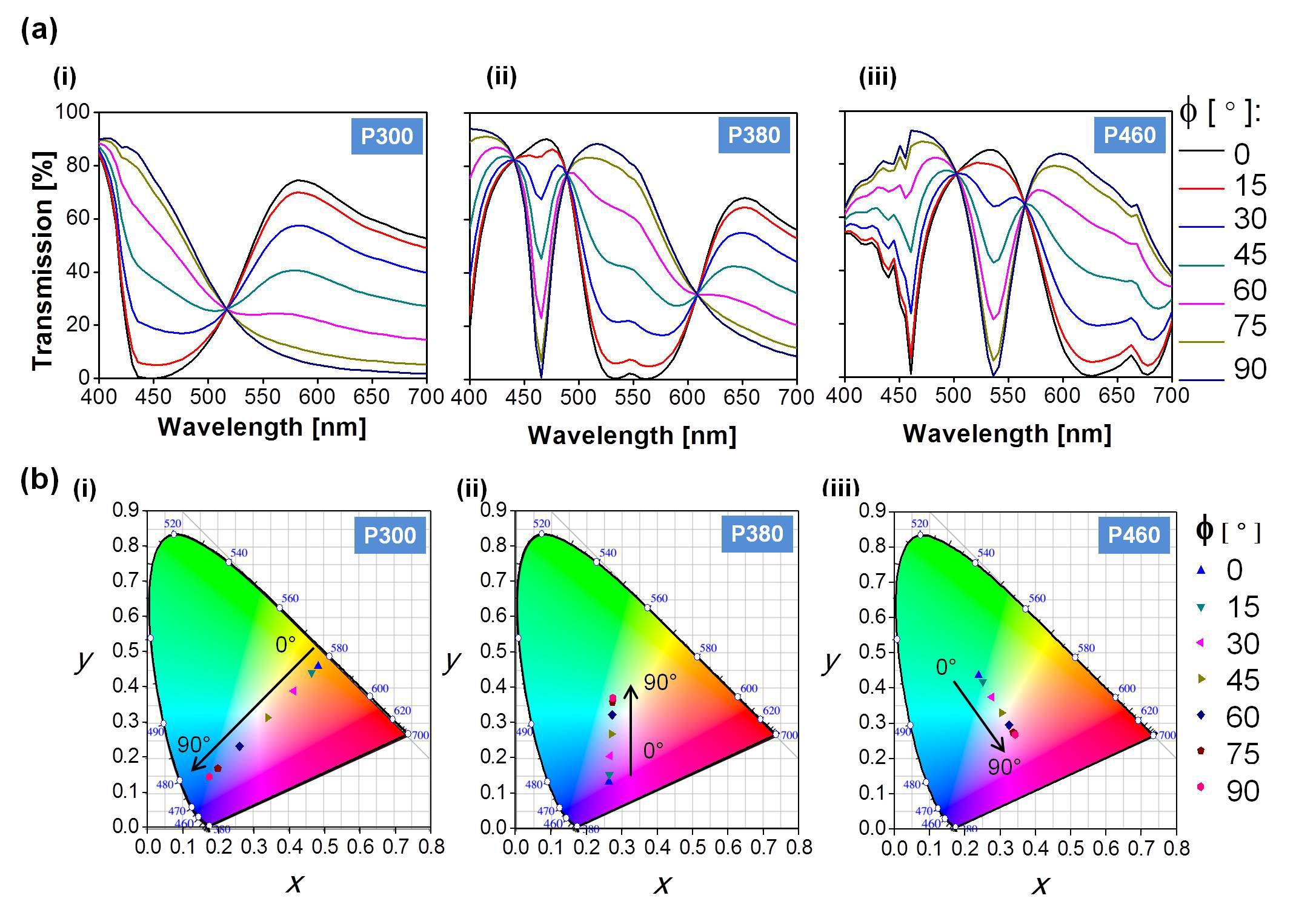
**

**Supplementary Figure S1.** (a) Simulated transmission spectra of the filters with periods of a NW array of (i) 300, (ii) 380 and (iii) 460 nm, respectively, from left to right, for polarization directions from ϕ=0 to 90° in steps of 15°. Transmission spectra were dependent on incident polarization hinting at the polarization-tailored tunability. (b) Chromaticity coordinates corresponding to the measured spectra for filters with periods of (i) 300, (ii) 380, and (iii) 460 nm in the CIE 1931 chromaticity diagram as the polarization angle ϕ varied from 0° to 90° in steps of 15°.

**
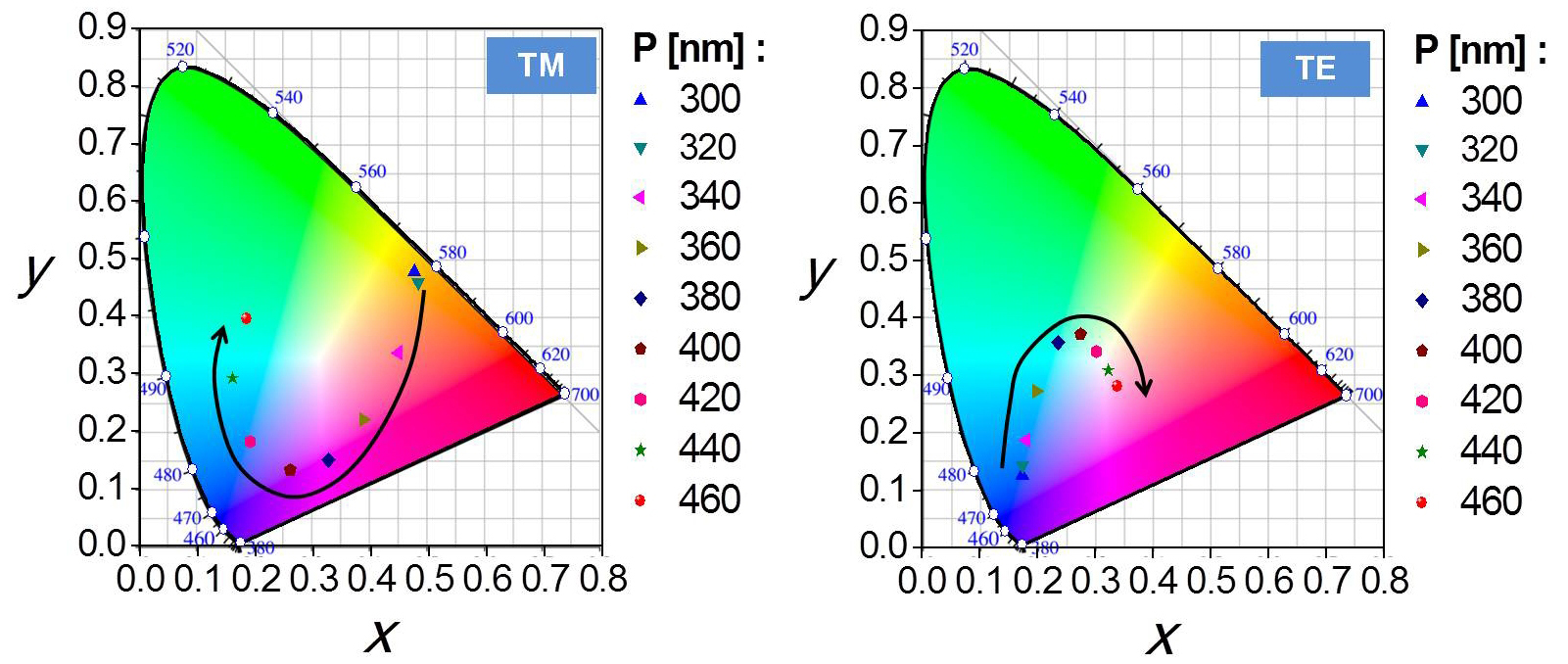
**

**Supplementary Figure S2.** Variations in the CIE 1931 chromaticity coordinates corresponding to the simulated transmission spectra for different periods of NW array for the TM and TE polarizations when the period was varied from 300 to 460 nm in steps of 20 nm.

**2.** **Optimization of the thickness of the dielectric overlay (H_d_):**

In order to determine the most appropriate thickness of the dielectric layer that could lead to an enhanced transmission for both TE and TM polarizations and different colors for the two polarizations, we attempted to check the transmission spectra and the corresponding color response by plotting the chromaticity coordinates in a CIE 1931 chromaticity diagram; we did this for the cases of a simple NW array atop a glass substrate and the cases where the slits were filled with a dielectric in conjunction with an overlay of different thicknesses under the two polarizations. We first took a simple case of an Al grating with a duty ratio of 0.5 and a thickness of 120 nm with no dielectric overlay. The transmission spectra and the corresponding chromaticity coordinates on a CIE 1931 chromaticity diagram are shown for different periods in Supplementary Figure 3. The transmission for the TE case was checked to be less than 50% while that, for the TM case, reached as high as 90%. For the TE case, only the blue color could be obtained irrespective of the variations in the period whereas, for the TM case, the transmitted color was around the yellowish color for different periods. Without the assistance of a dielectric overlay, we failed to realize any efficient transmission for both TE and TM polarizations and different colors depending on the period.

**
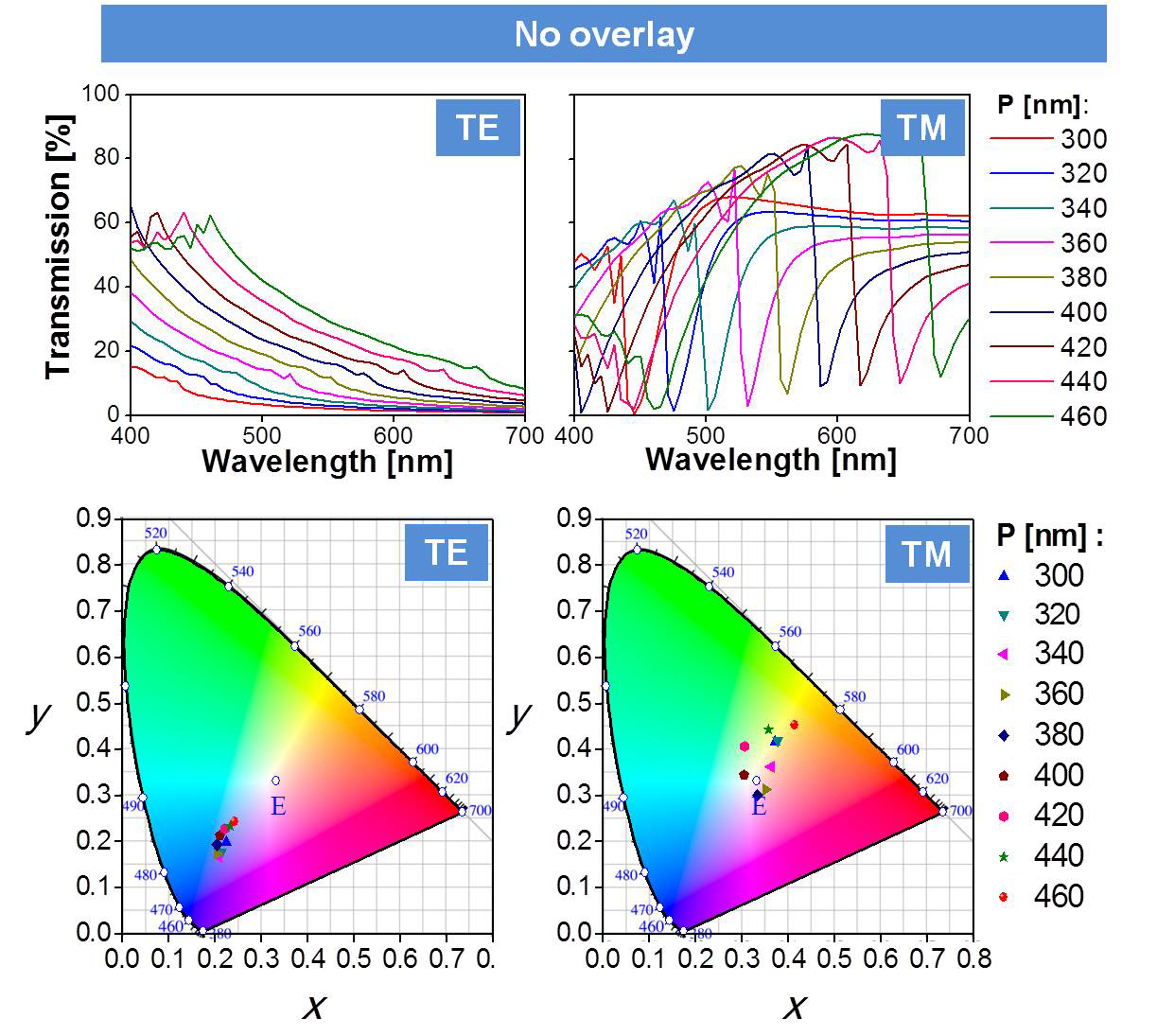
**

**Supplementary Figure S3**. Simulated transmission spectra of the devices with an Al NW array over a glass substrate and the corresponding chromaticity coordinates in the CIE 1931 chromaticity diagram for TE and TM polarizations as the period of the metallic NW array was varied from 300 to 460 nm. For the TE case, the transmission was below 50% and only the blue color could be obtained, regardless of the variations in the period; for the TM case, the transmission reached about 90% and the transmitted color was largely around the yellowish color for different periods.

Next, we filled up the slits between the Al NWs with SiO_2_, and we also introduced an SiO_2_ overlay of different thicknesses of H_d_ =100, 200 and 300 nm atop the Al NW and calculated the transmission spectra and corresponding chromaticity coordinates, which were plotted in a CIE 1931 chromaticity diagram for different periods P, as illustrated in Supplementary Figures S4, S5, and S6, respectively. The transmission for the two polarizations rose up to 90%. It was observed in all cases that the color output was dependent upon the period as well as the polarization, indicating a possibility of polarization-adjusted tuning. The thickness of the overlay was chosen to be H_d_=200 nm, in light of a wider hue of colors in accordance with the variation in the period.


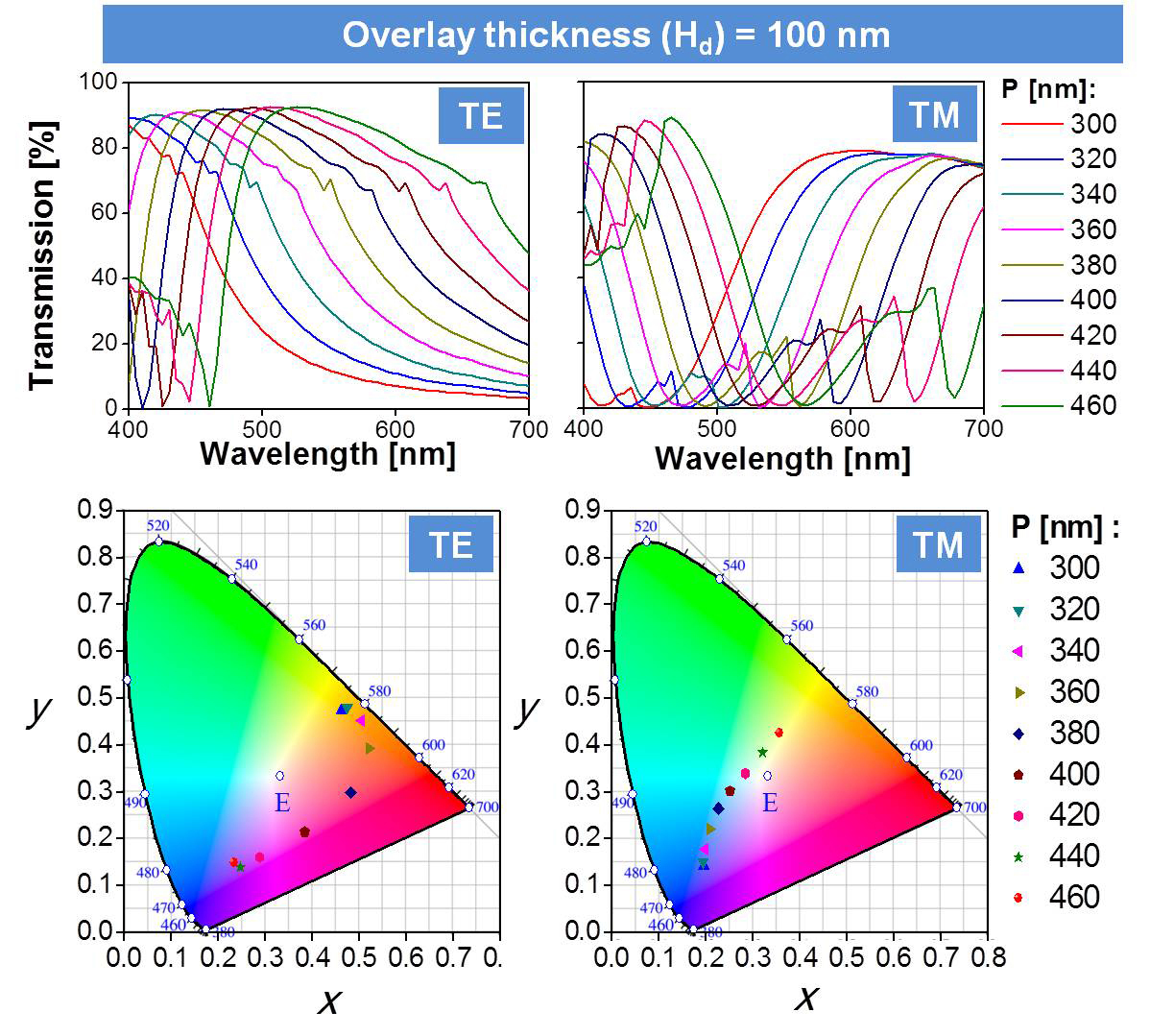


**Supplementary Figure S4.** Simulated spectra of the filters consisting of Al NWs, the slits of which were completely filled with SiO_2_ and were loaded with a SiO_2_ overlay of 100-nm thickness atop the NWs, along with the corresponding chromaticity coordinates in the CIE 1931 chromaticity diagram, for the TE and TM polarizations, when the period was varied from 300 to 460 nm.


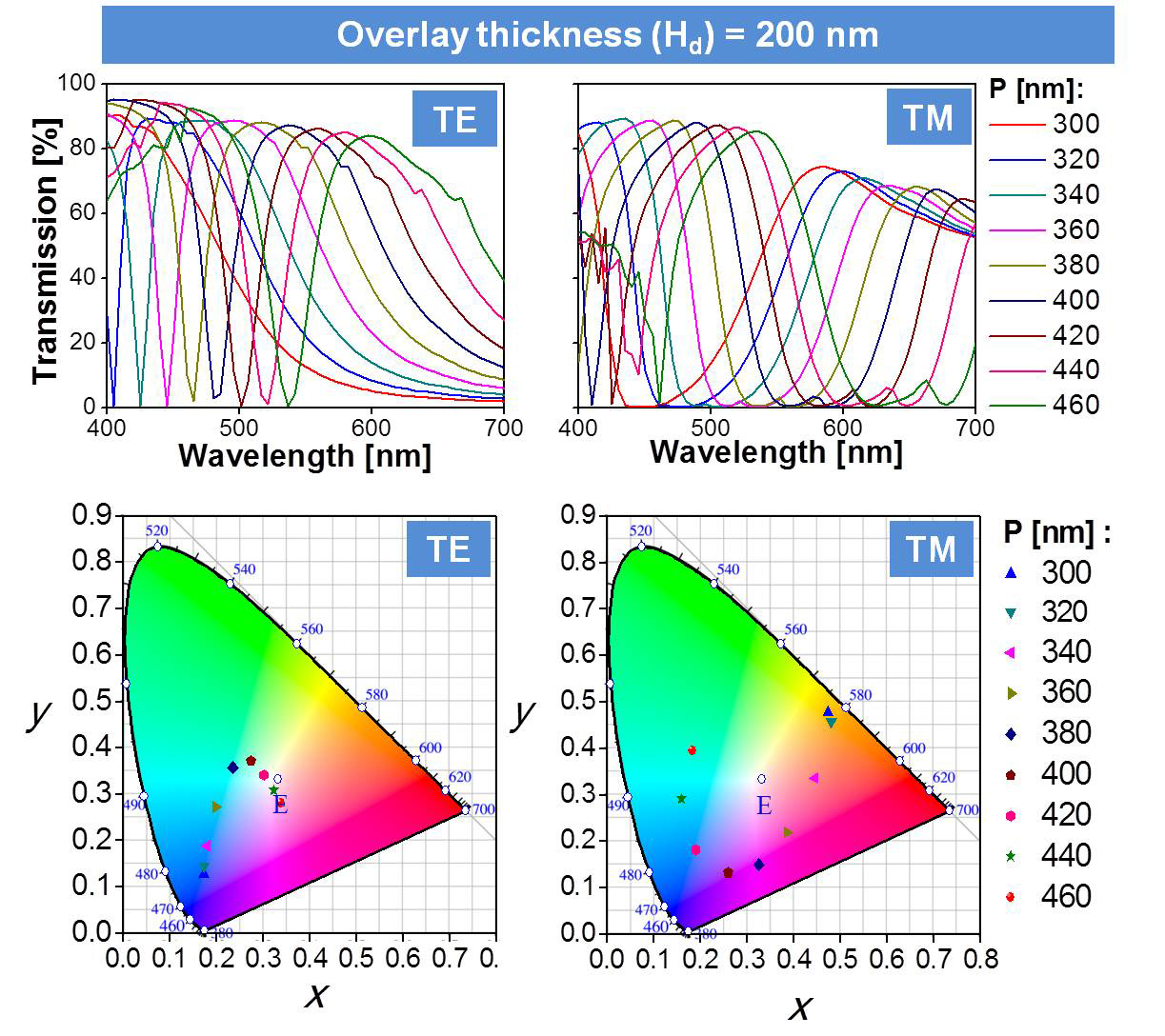


**Supplementary Figure S5.** Simulated spectra of the filters consisting of Al NWs, the slits of which were completely filled with SiO_2_ and were loaded with a SiO_2_ overlay of 200-nm thickness atop the NWs, along with the corresponding chromaticity coordinates in the CIE 1931 chromaticity diagram, for the TE and TM polarizations, when the period was varied from 300 to 460 nm.


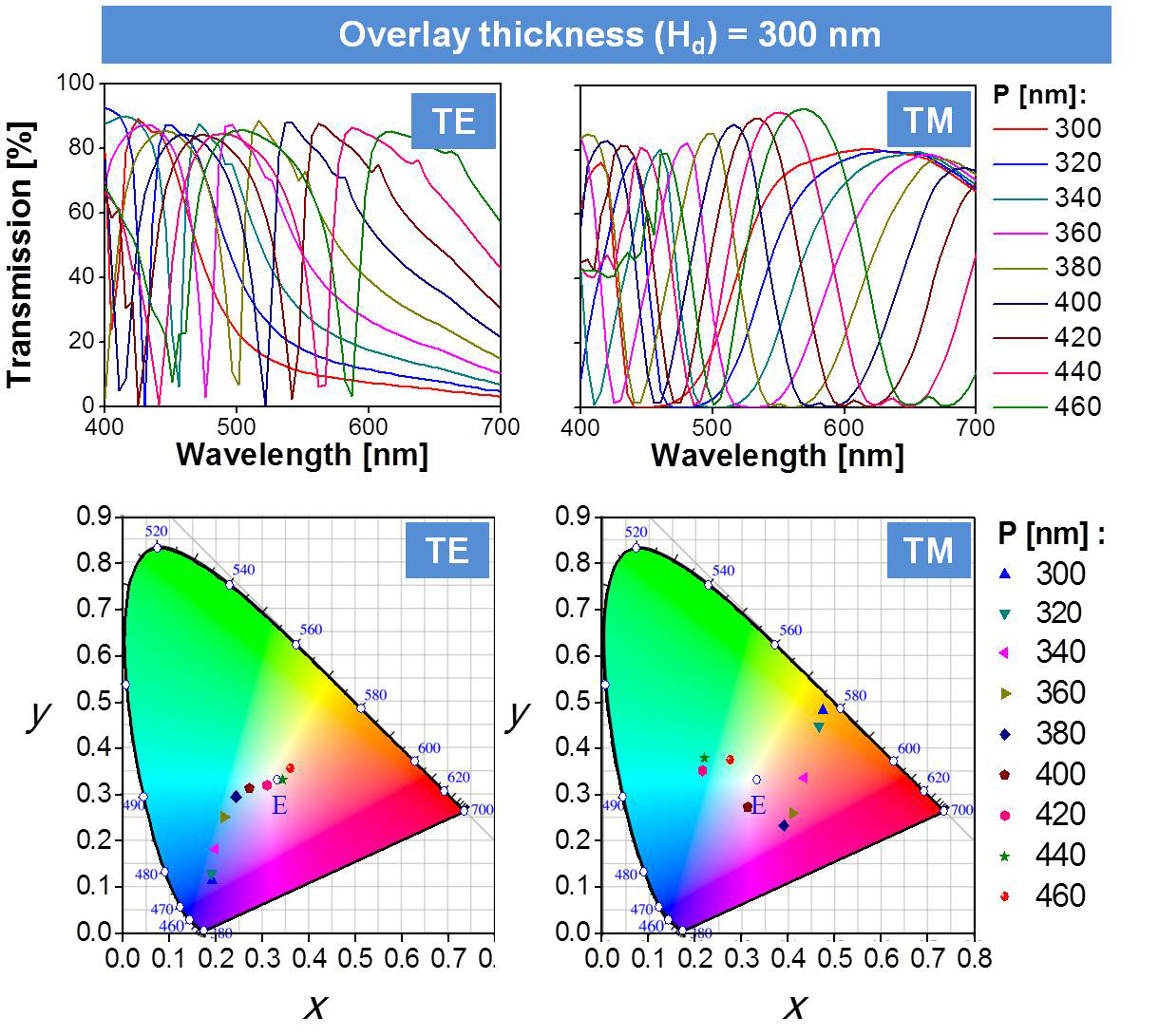


**Supplementary Figure S6**. Simulated spectra of the filters consisting of Al NWs, the slits of which were completely filled with SiO_2_ and were loaded with a SiO_2_ overlay of 300-nm thickness atop the NWs, along with the corresponding chromaticity coordinates in the CIE 1931 chromaticity diagram, for the TE and TM polarizations, when the period was varied from 300 to 460 nm.
